# Digital coaching and its potential to support the return-to-work-process for individuals with chronic musculoskeletal pain - A focus group study

**DOI:** 10.1177/20552076241300222

**Published:** 2024-11-18

**Authors:** Vedrana Bolic Baric, Gunilla Liedberg, Hanna Lundell, Mathilda Björk, Christina Turesson

**Affiliations:** 1 Department of Health, Medicine and Caring Sciences, Division of Prevention, Rehabilitation and Community Medicine, 4566 Linköping University, Linköping, Sweden; 2 Rehabilitation Medicine, Nyköping Hospital, Nyköping, Sweden; 3 Pain and Rehabilitation Centre, Department of Health, Medicine and Caring Sciences, 4566 Linköping University, Linköping, Sweden

**Keywords:** Chronic pain, employment, vocational rehabilitation, working conditions, qualitative research

## Abstract

**Background:**

Given the growing digitalization of healthcare and society, it becomes crucial to explore whether digital interactions with healthcare professionals, such as coaching, can offer effective support and contribute to an improved return-to-work process and a sustainable work environment for individuals with chronic musculoskeletal pain.

**Aim:**

To explore perceptions of digital coaching and its potential to support the return-to-work process for individuals with chronic musculoskeletal pain.

**Methods:**

Three focus group interviews consisting of 14 people—11 women and three men (with a mean age of 48 years)—were conducted. All participants had a goal of returning to work and had completed an interprofessional rehabilitation program due to chronic musculoskeletal pain. Data were analyzed using thematic analysis.

**Results:**

Findings show that integrating a coach into digital tools could offer new opportunities for personalized guidance, support and feedback to individuals during the return-to-work process. The first theme emphasizes the importance of sustained support throughout the entire return-to-work process—from rehabilitation programs to workforce integration. The second theme outlines the specific tasks and functions expected from a digital coach, as perceived by the participants. Lastly, the third theme explores the envisioned future evolution of digital coaching in chronic musculoskeletal pain management.

**Conclusions:**

Digital coaching offers promise in addressing challenges during the return-to-work process, acting as a bridge among stakeholders to ensure accessibility, continuity and coordination in rehabilitation and return-to-work efforts.

## Introduction

Chronic musculoskeletal pain (CMSP) (i.e., pain duration of more than three months), such as chronic neck/shoulder and back pain or generalized widespread pain (including fibromyalgia (FM)), are well-known issues with a prevalence ranging from 10.4% to 20% among adults.^1,2^ In CMSP, in addition to the pain itself, fatigue and concentration problems are often obstacles in everyday life.^3^ Living with CMSP can mean constantly having to deal with various symptoms that can lead to limitations in daily life, including work. For example, paid work and leisure activities may require more energy than before and necessitate long periods of recovery and rest to cope.^4,5^

Persistent pain presents a significant challenge, not only affecting individuals but also employers and society at large.^6^ People with CMSP often struggle to meet the demanding physical and mental requirements of the labor market, leading to sickness absence and presenteeism.^5^ Employees with CMSP report inadequate support from employers during the return-to-work (RTW) process. Simultaneously, employers lack knowledge on how to effectively assist employees with chronic pain.^7,8^ Continuous support from employers and other stakeholders is crucial for facilitating return-to-work and maintaining employment for individuals with chronic pain. This support aims to create a sustainable work environment that addresses current and future workplace challenges.^9^ Creating a sustainable work environment involves enhancing communication and interaction among relevant parties and implementing personalized work arrangements. Furthermore, self-efficacy, self-management techniques, and workplace demands and opportunities significantly impact the situation.^6,10,11^ Evidence-based information from qualitative experiences regarding CMSP and RTW demonstrates that, in addition to enhancing collaboration among stakeholders, active and consistent support among the employer and the individual plays a vital role in facilitating RTW. This support helps boost self-awareness, promotes changes in behavior and thinking, and strengthens the individual's capacity for self-adjustment. Furthermore, implementing tailored solutions that meet the specific needs of the individual in the workplace is essential for a successful RTW process.^6^

Digital coaching can be described as one way to support a person regarding necessary changes in lifestyle to improve self-management of chronic disease.^12^ It is a personalized process based on behavioral change theory where the coach applies a pedagogical approach to support the person in developing strategies to enhance the development of healthy habits.^13,14^ This process can be provided by healthcare staff, employers, or other stakeholders using different platforms and tools in various stages of the RTW process.^14^ Digital coaching uses a combination of technology and typically involves written communication through chat messages, but can also include video or virtual sessions, along with supplemental phone calls as needed.^15^ There is still a knowledge gap in how a digital coach should be designed.

Research regarding digital coaching to improve management of CMSP and continued support for self-management has investigated different solutions. Yates et al.^16^ for example revealed that digitally delivered peer support holds particular promise for individuals experiencing high levels of loneliness and coping with chronic non-cancer pain, particularly those with lower pain levels. Hausser-Ulrich et al.^17^ investigated the effect of a chatbot and found that while participants found the chatbot useful and enjoyable, the study did not uncover any significant changes in pain level or impairment. Further, Sander et al.^18^ investigated a web-based e-coach which showed promising results for preventing depression in people with low back pain. Thus, the concept of digital coaching has been used for different technological approaches. Digital tools for individuals managing chronic pain often lack the option to interact with healthcare professionals or other stakeholders, a key element recommended in self-care strategies for managing the condition.^19^ However, a research initiative involving individuals with CMSP, which included counseling sessions with healthcare professionals, showed a reduction in absenteeism and generated societal cost savings.^20^

With the increasing digitalization of healthcare and society, it is essential to investigate whether digital interactions with healthcare professionals, such as coaching, can provide effective self-management support and promote a sustainable work situation for people with disabilities, particularly those with CMSP.^19^ Technology, such as a digital coach during and following a rehabilitation program, for instance, has the potential to address obstacles like poor communication between stakeholders, inappropriate workplace adaptations and ineffective support.^21^ This can enhance the likelihood of people with CMSP returning to work or maintaining employment. Moreover, focusing on digital resources could help reduce the high societal costs associated with productivity loss due to chronic pain by mitigating current issues in the RTW process.^20^ Therefore, it is crucial to understand how individuals with CMSP view a digital coach and identify design elements that could make it effective for fostering a sustainable work situation. This study aimed to explore these perceptions and the potential of digital coaching to support the RTW process for individuals with CMSP.

## Method

### Study design

This study adopts a qualitative research design^22^ to capture the perceptions of digital coaching in supporting the RTW process for individuals with CMSP. Embedded in the research tradition of constructivism, this approach explores participants’ subjective experiences and perceptions within their societal contexts.^22,23^ The constructivist perspective helped researchers better understand the perceptions and potential benefits of digital coaching from the viewpoint of individuals with CMSP, based on their own RTW experiences. In 2022, three focus group interviews were conducted and analyzed with thematic analysis, following the steps of Braun & Clark.^23^ This article has been prepared in accordance with the consolidated criteria for reporting qualitative research (Appendix 1).^24^

### Participants and setting

Participants were recruited from two healthcare units in southern Sweden, located in Region Östergötland and Region Södermanland, which provide Interprofessional rehabilitation programs for individuals with CMSP. In Sweden, patients with CMSP may receive group-based Integrated Pain Rehabilitation Programs (IPRP) that focus on physical, psychosocial and work-related components, administered by multiprofessional teams. This specialized rehabilitation program is explicitly designed to facilitate the RTW process. According to the Swedish Agency for Health Technology Assessment and Assessment of Social Services (SBU) reports,^5^ IPRP improves the prospects of patients returning to work compared to either no intervention or less extensive interventions.

A relevance sampling strategy^22^ was applied with inclusion criteria specifying that participants must have completed IPRP due to CMSP within the last two years, possess the ambition to return to work and either have existing employment or be in the process of applying for employment. To ensure a diverse range of experiences we made an effort to include participants of different ages and genders. Individuals who did not speak or understand Swedish or had previously participated in a research project related to development of digital support for people with CMSP were excluded from the study.

Study participants from the two healthcare units were identified through the Swedish Quality Registry for Pain Rehabilitation (SQRP) and 50 people were contacted by phone during spring 2022. Twenty people declined participation at initial contact. Two people did not meet the inclusion criteria, five individuals were unreachable by phone and three declined participation due to scheduling conflicts that prevented them from participating on any available dates. Six individuals were excluded as the focus group had to be cancelled due to adverse weather conditions or late cancellation by the participants, and rescheduling the group was not possible. In all, 14 people consented to participate in the study — 11 women and three men with a mean age of 48 (range 36–62) years. The three focus groups consisted of three, five and six participants respectively. All participants were employed (Table 1).

**Table 1.** Background information of the study sample, n = 14.

Sex, n:	
Women	11
Men	3
Age, m (SD)	48 (8)
Years living with CMSP, m (SD)	12 (10)
Living status, n:	
Single person household	4
Married or cohabiting partner	10
Highest level of education, n:	
University	7
Upper secondary school	7
Employment: full-time/part-time, n	12/2
Sick leave, n:	
Full-time	3
Part-time	4
No sick leave	7
Sickness benefit, n:	
Part-time	1
None	13
Uses apps on mobile phone/computer/iPad, n:	
Daily/once	13
Several times per week	1
Prefers to use apps on, n:	
Mobile phone	4
Computer	9
Tablet, iPad	1

### Data collection

Data was collected through three digitally recorded focus group interviews, each lasting between 50 and 90 min. Pairs of researchers (GL & MB, GL & CT, HL & CT) facilitated each focus group, where one had a lead role and the other took notes. A semi-structured interview guide consisting of open-ended questions was developed by four of the authors (GL, MB, HL, CT), all of whom have significant experience with the target group, both in clinical practice and/or research. The interview guide (Appendix 2) contains questions about digital coaching, experiences of digital support during the RTW process, and desired RTW support. The interview guide was used to start and facilitate the group discussions. Both researchers asked follow-up questions as necessary.

Before the focus group discussion, participants filled in a questionnaire regarding background data. The research team developed the demographic questionnaire and pilot tested it with the assistance of colleagues from a pain clinic. These colleagues critically reviewed the questionnaire to ensure the questions were relevant to the target group and to identify any potential gaps. Based on their feedback, only minor adjustments were made, mainly simplifying the language.

### Context and researchers’ position

The focus group interviews were conducted at two different health care units and the researchers performing each of the three focus group interviews had no previous relationship with any of the study participants. The researchers introduced themselves with names and occupations before starting the focus groups.

### Analysis

**Figure 1.**
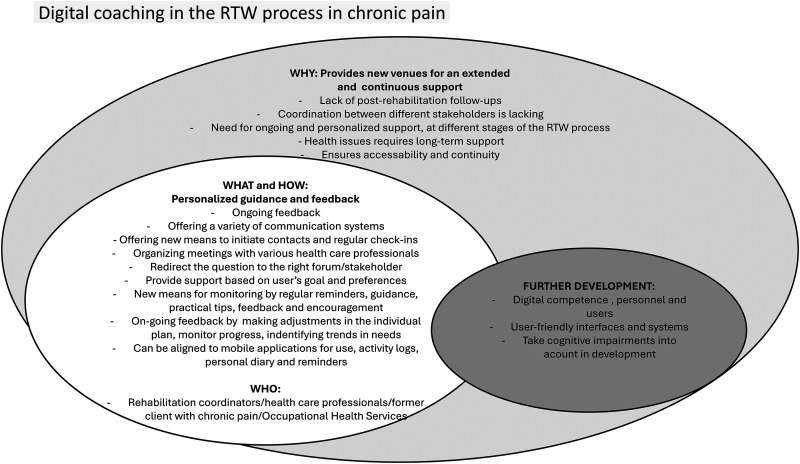
Digital coaching in the RTW process in chronic pain.

A thematic analysis following the steps of Braun and Clarke^23^ was applied to identify, analyze and report patterns or themes within data. In the first step of the analysis, the recorded interviews were transcribed verbatim. The transcribed focus groups were read several times by VB, CT and GL to achieve an overall understanding of the entire body of data or data corpus. Notes on early impressions were written down (e.g., that participants considered digital coaching as being synonymous with digital apps). The analysis proceeded to step two in which each segment of data that was relevant to the research question was coded. Open coding was used, whereby codes were developed and modified as VB worked through the coding process, rather than having pre-set codes.^23^ In step three, the codes were examined by GL and some were fitted together into preliminary themes. For example, several of the codes were related to the need for personal support that is continuous and prolonged throughout the RTW process. These codes were collected in an initial theme called “The need for digital support”. At the end of step three, all codes were organized into broader descriptive themes, with subthemes that described patterns in the data relevant to the research question. During step four, the preliminary themes were re-read and reviewed, modified, and further developed to ensure that the themes work in the context of the entire data set. The themes, subthemes and codes that emerged were all checked against each other and to the original data set before being discussed within the entire author group. Several subthemes have been revised. For example, the preliminary theme called “Digital coaching creates new opportunities for individual support” included codes that reflected different aspects of what the digital coach should do and how the support should be provided. We combined these into a new theme called “*Digital coaching for personalized guidance and feedback in the RTW process*, with two subthemes ‘what?’ and ‘how?’. During step five, final refinement of the themes was performed to identify the ‘essence’ of what each theme captures.^23^ All themes were re-read and short summaries of what is interesting about them and why were written down. Here it became evident that the qualities and background of the coach (i.e., ‘who’) needed further attention in terms of how this related to the other subthemes. It was jointly agreed to separate ‘who’ more clearly from the ‘what and how’ in the analysis. Thematic exhaustion, according to Braun & Clarke (2021),^25^ was achieved when no additional themes emerged from the data collection. All themes and subthemes were discussed with all authors before consensus was reached. Figure 1 is a final thematic map that illustrates the relationships between themes and subthemes.

### Trustworthiness

Credibility was attained by involving two authors (VB and GL) independently in the analysis. All authors were involved in discussions of the findings, critically questioning the first author's understanding of the text, and in the search for different patterns and alternative subthemes and themes, as suggested by Braun and Clark.^23^

Reflexive practices used during data collection and data analysis is one measure that enhances the dependability of qualitative research.^22^ All three pairs of researchers (GL & MB, GL & CT, HL & CT) participated in reflexive practices, prior to data collection and following each focus group interview. Further, VB maintained an audit trail of decisions made during analysis, as well as initial thoughts regarding codes and subthemes.

Confirmability of the results is addressed by providing a detailed description of the participants and the context, with the intention of enabling the reader to assess how transferable the results are to other contexts, as suggested by several authors.^22^

### Ethical considerations

This study complies with the Declaration of Helsinki.^26^ Participants were thoroughly informed about the study, assured that participation was voluntary and given the option to withdraw at any time. Written informed consent was obtained from all participants, and data confidentially was maintained through a secure database. The study was approved by the Swedish Ethical Review Board (Dnr 2021-07068-02).

## Results

In the focus group discussions, it was revealed that integrating a coach into digital tools could provide new opportunities for personalized guidance, support and feedback to individuals in the RTW process. The first theme, “*Digital coaching to meet the need for an extended and continuous support in the RTW process”* underscored the “*why”* in terms of sustained support throughout the entire RTW process, spanning from rehabilitation programs to integration into the workforce. The second theme, “*Digital coaching for personalized guidance, and feedback in the RTW process”* outlined the “*what”* in terms of envisioned tasks and functions “*how”* of a digital coach, as perceived by the participants. The third theme, “*Digital support in the RTW process - an area that needs further development”* delved into the “*future”* desired evolution of digital coaching in CMSP pain management, as identified by the participants.

### Digital coaching to meet the need for an extended and continuous support in the RTW process

The focus group discussions about digital coaching centered around challenges of post-rehabilitation follow-ups and coordination between different stakeholders in the RTW process. One participant used the analogy of “watertight compartments” (FG2, 62-year-old woman) to underscore the perceived barriers in the current return to work process, highlighting the lack of continuous support, which was considered a basic need by the participants. One participant stressed:

“*It's generally the case that one feels a bit lost, like being thrown to the wolves both before and after. What one might miss is precisely what you're looking for—coordinators and people who can be a bit of a spider in the web. Whether it's through a digital tool or happens through physical meetings at a healthcare center, I have no idea, but I miss having someone who keeps track of me. Because I don't always know where to turn.” (FG1, 50-year-old man)*

In addition to the need for ongoing support, there was also a need for individualized support in managing post-rehabilitation in the RTW process and sustaining employment. Despite their eagerness to work, participants shared difficulties in effectively managing disturbances in daily life caused by CMSP. For example, managing work tasks during regular hours often resulted in increased pain and the need for rest, limiting their ability to engage in family activities. Participants stressed that when working 100%, leisure time becomes about resting to save energy for working and “*how to make life function to be able to handle work,” (FG1, 43-year-old man)*. Although not visibly apparent, challenges related to CMSP hindered the RTW process:

“*Following a rehabilitation program, people assume that you're doing well. Everyone forgets that you're still unwell. That's been my experience. Now, I don't have support from my boss anymore. He perceives that I am working at 100%, so he can't comprehend that I'm still dealing with health issues, or perhaps he has forgotten about it. This is a bit tricky.” (FG1, 36-year-old woman)*

The importance of a support person such as a coach, especially during the phased increase in working hours, was emphasized:

“*It would be even better from the beginning, when you increase your working hours, whether it's from 25% to 50%, 50% to 75%, especially 75% to 100%. At that point, there should be some form of contact with the boss, like every three months or every other month; let's meet, so it's not on me to initiate it. Human Resources should be involved. Because at that point, you grit your teeth to manage the situation, since you don't have the energy for anything else, you can't handle this, and you skip asking for help with it.” (FG1, 43-year-old man)*

The advantage of integrating a coach early in the rehabilitation process was discussed in terms of providing opportunities for ongoing support, even after leaving rehabilitation programs for ensuring accessibility and continuity of rehabilitation. The participants highlighted the need for a coach to be involved through the entire RTW process, from before leaving rehabilitation programs, through preparing for return to work, and then when back at work, which could contribute to a feeling of support and connection:

“*And that's what it is, and where digital tools might be helpful right from the start. That is, you go in and work in an app and you have this feedback even afterwards…I think it could help and then it becomes a gateway in some way that you can continue, maybe until you get back to work… They (the healthcare professionals) say that they will take care of the patient forever, that's not really true, for me anyway.” (FG3, 57-year-old-man)*

### Digital coaching for personalized guidance and feedback in the RTW process

A digital coach was specifically described by participants as a potential way for providing them with ongoing feedback, better communication about post-treatment plans and guidance in the RTW process, using different tools such as chat interfaces, video calls, messaging systems or mobile applications. The participants highlighted the potential that a digital coach brings for initiating contact and organizing meetings with various healthcare professionals, which is important for improving handovers and ensuring seamless communication between stakeholders. Also, a digital coach could redirect the question to the right forum or stakeholder.

Regarding how digital coaching could be implemented, rehabilitation coordinators were specifically mentioned as potential digital coaches. However, the question of healthcare professionals serving as digital coaches was discussed, with some participants highlighting that a coach should have some form of healthcare competence, while others suggested that a digital coach could have more general expertise, as individuals may need assistance beyond medical support. This includes emotional support and practical help with work-related issues. This kind of support could be provided by someone with personal experience, such as a former client with CMSP. However, participants stressed that this individual should have progressed somewhat in their RTW process to offer adequate support, even though this opinion was not shared by all:

“*I feel it wouldn't work because the person must be a healthy patient, not ill… However, having someone completely equivalent might end up causing more harm.” (FG3, 57-year-old-man)*

The added value of having a digital coach was discussed in terms of someone that could provide invaluable personalized support and strategies tailored to the individual's goals and preferences. These plans could include workout routines, meal plans, mindfulness exercises or any other activities aligned with the individual's goals. The coach could provide regular check-ins or reminders, guidance on daily routines and practical tips such as taking breaks, going for a walk, stretching or practicing deep breathing that relates to individual plans:

“*But if there is some kind of online coach, maybe: ´Well, how does your day look? When do you get up in the morning? Do you eat breakfast? Do you have any breaks? Can you take a walk? What do you need? Can you stand up and stretch for a while? Can you tell everyone, now we take five’. It can be such small things in the end that make it so…´Can you breathe deeply for 3 min every hour?’.” (FG1, 43-year-old man)*

When discussing how digital coaching could function, participants considered a digital coach as someone who could be more readily available than traditional paths to healthcare professionals, through various communication channels used at different steps of the RTW process. Real-time communication with a digital coach could occur using, for instance, chat interfaces and live chats or video calls, preferable for providing more context or explanation, and answers to be received immediately from someone familiar with pain. Digital question boxes or e-mails could, on the other hand, be used for non-urgent matters:

“*A digital question box works quite well if, in the best-case scenario, you try to describe your problem and someone gets back to you. Instead of waking up every morning to call and stand in line and maybe get something for the healthcare center… It would be nice to be able to send an e-mail. Can someone contact me regarding this matter I have?” (FG1, 43-year-old man)*

Integrating a coach into mobile applications was discussed in terms of offering new opportunities to store data that could be used by coaches for providing participants with personalized guidance, support and feedback. Mobile apps were discussed in terms of providing opportunities for integrating tools for tracking user progress over time, such as activity logs, personal diaries and reminders. The participants stressed that these tools could be analyzed by a coach to provide feedback, encouragement and motivation to individuals and adjust the user's plan as needed. As such, individualized data could be used to monitor progress, identifying trends in needs and behavior, setting and tracking personal goals and based on that provide support:

“*Yes, reminders of what tools you took with you. Then it becomes personal for oneself… but still. It may sound a bit simple, perhaps, but sometimes you actually forget that or remember that we talked about this, think like this. So that you can lean on that a bit, to go back and just update. To feel a bit at home in it, or whatever you want to call it. I think that would be very important.” (FG3, 48-year-old woman)*

However, none of the participants had experience of such an app being used in the RTW process or CMSP management. Suggested features and functionalities to be implemented into a mobile app from the participants were based on earlier experiences of using specialized apps within other areas such as cancer care or fitness, not within CMSP management.

### Digital coaching in the RTW process - an area that needs further development

Despite recognizing the added value of having a digital coach available for check-ins and offering personalized strategies in the different steps of the RTW process, the participants also discussed reasons for the limited uptake of technology-based solutions for CMSP in the RTW process. The need for digital competence and the importance of both personnel and users’ digital proficiency in learning to use technology were described as influencing uptake of digital tools in the RTW process. Participants further discussed cognitive impairments associated with CMSP and the challenges individuals may experience when navigating complex digital tools used for digital coaching. In particular, user-friendly interfaces and systems were described as important for ensuring that anyone, regardless of technical expertise, can navigate the tools effortlessly.

Several concerns were also raised about the impersonal nature of digital communication with the coach. Therefore, digital communication systems should complement rather than replace human interaction. Also, participants expressed the need for technically stable systems, without technical hiccups, such as unstable connections with Zoom meetings that may hinder communication:

“*Yeah, that can definitely happen, you know, just trying to get everything to run smoothly…But, like I was saying, with these Zoom meetings, it doesn't always go smoothly, you know? Like, ‘Wait, we can't hear anything,’ and then you're scrambling to figure out what's wrong, and sometimes, you just can't fix it. Yeah, technical issues can be a real challenge.” (FG2, 50-year-old woman)*

## Discussion

The aim of this study was to explore the perceptions of digital coaching and its potential to support the RTW process. Overall, it became evident that the participants lacked continuous support in their RTW process and expressed a desire for continuous support, not only with their work but also in interactions with their employer and the Swedish Social Insurance Agency (SSIA).

The findings concerning the concept of digital coaching in terms of “*why”* emphasized the critical need for extended and continuous coaching support beyond formal rehabilitation programs from employers and other stakeholders in facilitating RTW and maintaining employment among individuals with CMSP. The suggested value of digital coaching in the RTW process is evident from the challenges highlighted by participants including a sense of being abandoned or lost, both before and after rehabilitation due to a perceived absence of continuous support. Further, managing everyday challenges in dealing with CMSP and balancing work and leisure requires someone to coordinate and monitor individuals’ progress consistently. These reported challenges are all in line with earlier studies^6,21^ highlighting the significance of extended collaboration among stakeholders, the importance of balancing work, leisure, family and health and tailored support at the workplace for sustained employment.^6,27,28^ What is interesting and in line with recent research^29^ is the potential that digital coaching brings in serving as a bridge between stakeholders (e.g., individuals, healthcare providers, employers) to ensure accessibility, continuity and coordination in the RTW process.

The concept of digital coaching in terms of “*how”* was described as providing new means for ongoing engagement throughout the RTW process, offering personalized guidance and feedback, in line with earlier studies.^20^ Participants in this study emphasized the importance of regular contact and structured interactions with employers and Human Resources (HR) to manage expectations, especially during the phased increase in working hours. Findings revealed that integrating digital coaching early in the RTW process ensures regular check-ins, facilitates ongoing and enhanced communication about post-treatment plans with stakeholders and improves coordination facilitating a phased return to work. This is especially significant since previous research underscores the benefits of a comprehensive RTW plan tailored to individual needs and developed by and anchored with all involved stakeholders, as a key factor for facilitating a RTW.^6,7,30^ Additionally, clearly defined roles and responsibilities for stakeholders involved, along with effective communication and collaboration during the rehabilitation process, can enhance RTW.^6,30^ Therefore, strategies that ease and find efficient ways for stakeholders to collaborate must be prioritized for a successful RTW.

Findings in the present study show that a digital coach is perceived as someone more readily available than traditional paths to gain contact with healthcare professionals, utilizing various communication channels throughout the RTW process. Real-time communication methods were highlighted in the current study as valuable for providing immediate context, explanations and answers from someone familiar with pain management. Earlier studies^31,32^ show that synchronous communication, which involves real-time, face-to-face coaching (image and voice) are more user-friendly and cost-effective in terms of lower travel costs for both individuals and healthcare providers and reduced unscheduled visits. However, findings in the current study also show that digital question boxes or e-mails could, on the other hand, be used for non-urgent matters. The benefits of asynchronous communication, in which monitoring and feedback is delivered via e-mail, automated messaging systems or other tools without face-to-face contact, have been recognized by earlier studies^31^ for allowing time for reflection due to delayed response times. Taken together, our findings - as well as earlier studies^31,32^ - point to the need for further research to explore how synchronous and asynchronous communication can be used as integrated systems of modern smartphones or other smart technology for providing monitoring and feedback during the RTW process.

Additional results concerning the “*how”* emphasize that digital coaching in CMSP pain management is a promising approach for improving personalized support and delivering tailored strategies and guidance aligned with individuals’ unique goals and preferences. Moreover, a digital coach has the potential to customize support based on individual needs, such as providing strategies to manage work-related activities while addressing challenges like CMSP. This approach promotes sustainable employment and work-life balance, as suggested by prior research.^6^ However, findings concerning the “*who”* indicate that there is a diversity of opinions on the qualifications and backgrounds of digital coaches. For rehabilitation and CMSP management, coaches with a background in healthcare can offer valuable insights and guidance that are medically informed and tailored to individual patient needs, in line with earlier studies.^31,33^ On the other hand, individuals undergoing RTW often require more than just medical advice. Emotional support, coping strategies and practical assistance with work-related issues are crucial aspects of their RTW journey, and as such digital coaches should be equipped to address these multifaceted needs effectively and represent stakeholders with diverse and additional expertise. Further, it is emphasized that former clients who have successfully navigated similar challenges can offer unique perspectives and empathetic support, lending credibility and practical insights to their guidance. The role of a rehabilitation coordinator as a coach was also mentioned. It has been shown that a rehabilitation coordinator is a success factor for a faster and better return to work experience as they collaborate internally and with external stakeholders.^6,34^ Further research should consider these varied perspectives to ensure that coaching programs are inclusive, effective and aligned with the specific needs and preferences of individuals undergoing RTW. Findings concerning “*future needs”* of digital coaching, regarding the limited uptake of technology-based solutions for CMSP in the RTW process, stress the need for all involved- coaches, healthcare personnel, and users- to have adequate digital competence for the successful adoption and utilization of technology-based tools in CMSP management and RTW support.

This is especially important since findings suggest that stakeholders’ and users’ limited proficiency with technology-based tools and cognitive impairments associated with CMSP might limit the uptake of digital tools during RTW.^35^ Previous reviews^36–38^ highlighted a lack of usability and accessibility of available pain-management apps for individuals with CMSP and diverse needs, emphasizing the necessity for increased user and stakeholder involvement in the development of digital platforms and apps. Findings in the present study are in line with previous research^36–38^ that points to the need for personalized support throughout the RTW process, and particularly after formal rehabilitation programs.^7^ However, few existing pain self-management apps incorporate features related to personalized goal setting and consultation with healthcare professionals.^36,37^ Earlier studies^31^ have stressed the need for coaches to assist individuals in setting realistic, short-term and measurable subgoals to ensure distant goals do not decrease the individual's self-efficacy. Notably, participants expressed a desire for ongoing coaching throughout the RTW process, posing challenges related to time and resources. Addressing how to sustain personalized coaching over time warrants further investigation and this topic represents a valuable area for future research. Given the evolving nature of technology and rehabilitation needs over time, continuous improvement and adaptation of digital tools based on user feedback and technological advancements are necessary. This ensures that digital coaching solutions remain relevant, effective and responsive to the evolving challenges faced by individuals in the RTW process.

## Strengths and limitations

To the best of our knowledge, this study is the first that identifies how people with chronic pain described their understanding of a digital coach and what such a function could mean to them in their RTW process.

The study sample consists of people recruited from two specialist care units providing IPRP in Sweden and the ability to transfer these findings to other clinics are limited. The selection from IPRP clinics was made because these participants have relevant experiences for the study's topic. IPRP has been shown to improve the prospects of people returning to work, compared to either no intervention or less extensive intervention.^5^ The sample is predominantly composed of women, which reflects the clinical demographics of CMSP patients treated with IRPR in Sweden.^39^ The sample includes few men and lacks younger adults, which, along with the small number of participants, makes the results difficult to transfer to men as well as the youngest part of the CMSP population. Additionally, recruiting from specialist units means participants have more complex conditions and different RTW experiences, which may not represent the entire CMSP population. Therefore, the findings of this study are specific to its context and cannot be assumed to apply to all individuals with CMSP.

Nevertheless, the participants, procedure and data analysis have been thoroughly described to facilitate transferability.^22^ The ideal size for a focus group is between five and eight participants, although groups of four to six have become more common.^40^ Despite one group consisting of only three participants, the three focus-group interviews conducted in this study provided rich data.

The results should be interpreted considering the constructed dynamics of a focus group setting. For instance, the most articulate individuals may be more likely to participate in focus groups,^40^ and group dynamics can be disproportionately influenced by those who are more vocal or comfortable speaking in group settings, potentially affecting the transferability. Group dynamics can influence responses, and participants in such interviews may be more articulate.^40^ A potential risk in group interviews is that participants might respond in a socially desirable manner, introducing bias. To fully capture participants’ opinions, it could have been beneficial to have them write down their views of coaching and the support they needed before the group discussion, a method recommended alongside focus group methodology.^40^ Nonetheless, the focus group interviews facilitated in-depth discussions and captured a diverse array of viewpoints. The participants demonstrated commitment, and their familiarity and experience with their own RTW processes were deemed adequate.

At the end of each focus-group, the participants were shown four examples of how digital coaching could be provided. This was a more directed part of the focus group interviews, and it was a conscious choice to introduce these examples at the end to allow participants to reflect and discuss the topic of digital coaching freely first. Although these examples may not cover all possible solutions for providing digital coaching, they provided a foundation for elucidating the participants’ opinions of the topic further and gave valuable information about their experiences and preferences.

The researchers leading each focus group interview had no prior knowledge of the participants or the unit from where they were recruited. However, they possessed clinical or research experience with CMSP, RTW processes and digital support applications, which was valuable for facilitating discussions. To ensure consistency,^40^ the moderators participated across different groups. A notable strength of this study was the collaborative analysis process involving multiple researchers. The themes and subthemes were reviewed several times to assure they were grounded in the data.

## Conclusions and clinical implications

The findings underscore the critical importance of extended and continuous support beyond formal rehabilitation programs for individuals with chronic pain. Healthcare providers and employers should collaborate to provide ongoing support to facilitate sustained employment and successful RTW. Participants expressed feeling abandoned or lost before and after formal rehabilitation programs due to a lack of continuous support. Digital coaching shows promise in addressing the challenges faced by individuals with chronic pain during the RTW process. It may serve as a bridge between stakeholders (individuals, healthcare providers, employers) to ensure accessibility, continuity and coordination in rehabilitation and RTW efforts. Effective communication and collaboration among different stakeholders (healthcare providers, employers, HR, social insurance agencies) are essential for successful RTW. Digital coaching can facilitate this collaboration by initiating contact, scheduling meetings and ensuring the right stakeholders are involved. Digital coaches were described as “spiders in the web” with rehabilitation coordinators specially suggested as potential coaches. Digital coaching also allows for personalized support and tailored strategies aligning with individual goals and preferences. This approach promotes sustainable employment and work-life balance, which are crucial for individuals managing chronic pain. While digital coaching offers benefits such as improved accessibility and communication, challenges such as digital competence exist. There is a need to further enhance the usability and accessibility of digital tools for individuals with chronic pain, ensuring they can effectively engage with technology-based support during RTW. Given the claim that technology is a promise for future rehabilitation, more research is needed from different perspectives, including employees at different stages of the RTW process, employers, supervisors, and significant others, to investigate the role of digital coaching in optimizing RTW, providing individualized support and monitoring results, and deepening collaboration between stakeholders involved in the RTW.

## Supplemental Material

sj-docx-1-dhj-10.1177_20552076241300222 - Supplemental material for Digital coaching and its potential to support the return-to-work-process for individuals with chronic musculoskeletal pain - A focus group study Supplemental material, sj-docx-1-dhj-10.1177_20552076241300222 for Digital coaching and its potential to support the return-to-work-process for individuals with chronic musculoskeletal pain - A focus group study by Vedrana Bolic Baric, Gunilla Liedberg, Hanna Lundell, Mathilda Björk and Christina Turesson in DIGITAL HEALTH

sj-docx-2-dhj-10.1177_20552076241300222 - Supplemental material for Digital coaching and its potential to support the return-to-work-process for individuals with chronic musculoskeletal pain - A focus group study Supplemental material, sj-docx-2-dhj-10.1177_20552076241300222 for Digital coaching and its potential to support the return-to-work-process for individuals with chronic musculoskeletal pain - A focus group study by Vedrana Bolic Baric, Gunilla Liedberg, Hanna Lundell, Mathilda Björk and Christina Turesson in DIGITAL HEALTH

## Abbreviations

CMSP: Chronic musculoskeletal pain
FG: Focus group
FM: Fibromyalgia
HR: Human Resources
SQRP: Swedish Quality Registry for Pain Rehabilitation
RTW: Return-to-work
SBU: Swedish Agency for Health Technology Assessment and Assessment of Social Services
SSIA: Swedish Social Insurance Agency
