# Supplementary material for: Digital coaching and its potential to support the return-to-work-process for individuals with chronic musculoskeletal pain - A focus group study
Source: Digit Health. 2024 Nov 18;10:20552076241300222. doi: 10.1177/20552076241300222 (PMC11571250; doi:10.1177/20552076241300222)
Supplement: sj-docx-1-dhj-10.1177_20552076241300222 - Supplemental material for Digital coaching and its potential to support the return-to-work-process for individuals with chronic musculoskeletal pain - A focus group study [file sj-docx-1-dhj-10.1177_20552076241300222.docx]

| Appendix 1. Consolidated criteria for reporting qualitative studies (COREQ): 32-item | | |
| --- | --- | --- |
| No Item | Guide questions/description | Study description |
| Domain 1: Research team and reflexivity | | |
| *Personal Characteristics* | | |
| 1. Interviewer/facilitator | Which author/s conducted the interview or focus group? | Pairs of researchers (Gunilla Liedberg & Matilda Björk, Gunilla Liedberg & Christina Turesson, Hanna Lundell & Christina Turesson) facilitated each focus group. |
| 1. Credentials | What were the researcher’s credentials? | Baric holds a PhD and is a senior lecturer. Liedberg holds a PhD and an Associate Professorship (swe: Docent). Lundell is a registered Occupational Therapist with a master’s degree. Turesson holds a PhD and is an Associate Professor. Björk is a Professor, all in Occupational Therapy. |
| 1. Occupation | What was their occupation at the time of the study? | Baric, Liedberg, Turesson and Björk are all employed at Linköping university. Lundell is an Occupational therapist employed at the Department of rehabilitation medicine at Nyköping Hospital. |
| 1. Gender | Was the researcher male or female? | All were female. |
| 1. Experience and training | What experience or training did the researcher have? | The research group have significant experience with the target group, both in clinical practice and research. Additionally, the research group has a strong background in conducting qualitative studies and creating digital tools. |
| *Relationship with participants* | | |
| 1. Relationship established | Was a relationship established prior to study commencement? | The participating healthcare units were identified through the Swedish Quality Registry for Pain Rehabilitation (SQRP). Participants were contacted by the phone. |
| 1. Participant knowledge of the interviewer | What did the participants know about the researcher? *e.g. personal goals, reasons for doing the research* | All participants received written information about the study aim and procedure before giving written consent to participate. All participants were then informed again about the aim and procedure of the focus groups at the beginning of each focus group, both in writing and verbally. No personal information other than names and occupation were provided. |
| 1. Interviewer characteristics | What characteristics were reported about the interviewer/facilitator? | Pairs of researchers (GL & MB, GL & CT, HL & CT) facilitated each focus group. No personal information other than names and occupations were provided. |
| Domain 2: study design | | |
| *Theoretical framework* | | |
| 1. Methodological orientation and Theory | What methodological orientation was stated to underpin the study? | Thematic Analysis (as described by (Braun & Clarke, 2006). |
| *Participant selection* | | |
| 1. Sampling | How were participants selected? | A relevance sampling strategy was applied with inclusion criteria (participants must have completed IPRP due to CMSP within the last two years; possess the ambition to return to work; and either have existing employment or be in the process of applying for employment. Participants from two healthcare units were identified through the Swedish Quality Registry for Pain Rehabilitation (SQRP). |
| 1. Method of approach | How were participants approached? | Participants were contacted by telephone. Focus group interviews were conducted face-to-face at the health care units. |
| 1. Sample size | How many participants were in the study? | The three focus groups (n=14) consisting of three, five and six participants respectively. |
| 1. Non-participation | How many people refused to participate or dropped out? Reasons? | 50 people were contacted by telephone. Twenty people declined participation at initial contact. Two people did not meet the inclusion criteria, five individuals were unreachable by telephone and three declined participation due to scheduling conflicts. One focus group with six participants had to be cancelled due to adverse weather conditions. |
| *Setting* | | |
| 1. Setting of data collection | Where was the data collected? | Focus group interviews were conducted at the two health care units. |
| 1. Presence of non-participants | Was anyone else present besides the participants and researchers? | No |
| 1. Description of sample | What are the important characteristics of the sample? | Women /Men: 11/3  Age, m (SD): 48 (8)  Years living with CMSP, m, SD: 12 (10)  Single person household/ Married or cohabiting partner: 4/ 10  Employment full/part-time: 12/2 |
| *Data collection* | | |
| 1. Interview guide | Were questions, prompts, guides provided by the authors? Was it pilot tested? | The interview guide has been added as an appendix. |
| 1. Repeat interviews | Were repeat interviews carried out? If yes, how many? | No. |
| 1. Audio/visual recording | Did the research use audio or visual recording to collect the data? | Audio recordings were made of all focus groups. |
| 1. Field notes | Were field notes made during and/or after the interview or focus group? | Yes, mainly for follow-up questions. The analysis relies on the transcribed recordings. |
| 1. Duration | What was the duration of the interviews or focus group? | Between 50 and 90 minutes. |
| 1. Data saturation | Was data saturation discussed? | Thematic exhaustion, according to Braun & Clarke (2021), was achieved when no additional themes emerged from the data collection. |
| 1. Transcripts returned | Were transcripts returned to participants for comment and/or correction? | No. |
| Domain 3: analysis and findings | | |
| *Data analysis* | | |
| 1. Number of data coders | How many data coders coded the data? | 4 (VB, GL, HL, CT) |
| 1. Description of the coding tree | Did authors provide a description of the coding tree? | Yes, examples of codes, initial themes, subthemes and themes are provided. |
| 1. Derivation of themes | Were themes identified in advance or derived from the data? | Themes were derived from the data. |
| 1. Software | What software, if applicable, was used to manage the data? | NA. |
| 1. Participant checking | Did participants provide feedback on the findings? | No. |
| *Reporting* | | |
| 1. Quotations presented | Were participant quotations presented to illustrate the themes / findings? Was each  quotation identified? | Quotes are presented and identified by focus group, male/female and age. |
| 1. Data and findings consistent | Was there consistency between the data presented and the findings? | Yes. |
| 1. Clarity of major themes | Were major themes clearly presented in the findings? | Yes, both in the manuscript under the heading “Analysis” and “Results” and in Figure 1. |
| 1. Clarity of minor themes | Is there a description of diverse cases or discussion of minor themes? | Yes, both in the manuscript under the heading “Analysis” and “Results” and in Figure 1. |
