# Supplementary material for: Digital coaching and its potential to support the return-to-work-process for individuals with chronic musculoskeletal pain - A focus group study
Source: Digit Health. 2024 Nov 18;10:20552076241300222. doi: 10.1177/20552076241300222 (PMC11571250; doi:10.1177/20552076241300222)
Supplement: sj-docx-2-dhj-10.1177_20552076241300222 - Supplemental material for Digital coaching and its potential to support the return-to-work-process for individuals with chronic musculoskeletal pain - A focus group study [file sj-docx-2-dhj-10.1177_20552076241300222.docx]

**Appendix. 2 Interview guide**

Introduction: Today, we will explore and reflect on the potential benefits of ongoing support from a digital coach in your return-to-work process (RTW). We are interested in hearing your thoughts on what this support could entail for you personally, and in what ways you believe it could assist or make a difference in your return to work.

**Explore Perceptions:**

- What comes to mind when you hear the term "digital coaching"? What do you associate it with?

**Explore Experiences:**

- • How did you find the support for your return to work after completing your pain rehabilitation?
  • What aspects of the support were helpful, and what areas did you feel could have been improved?

**Explore the Following Areas Further:**

- What would you want a digital coach to support you with?
- In what situations or at what times would digital coaching be relevant for you?
- How do you think digital coaching should operate or be structured?
- Who do you envision as a suitable digital coach?
- What role or significance would a digital coach have for you after completing pain rehabilitation?
- If you don’t feel the need for a digital coach: What are the reasons behind your lack of interest?

Provide participants with **various examples** of what a digital coaching service could look like:

| 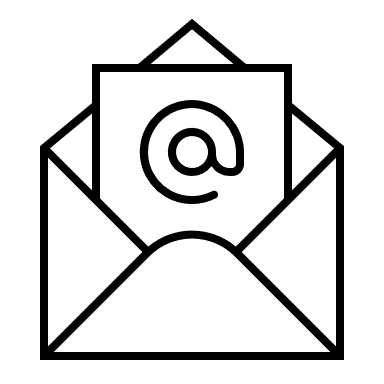 | Question Box: You can submit a question to the coach and receive a digital response within a week. |
| --- | --- |
| 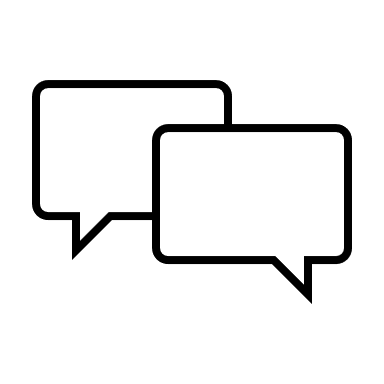 | Live chat: You have the opportunity to chat with a coach on certain days/times. |
| 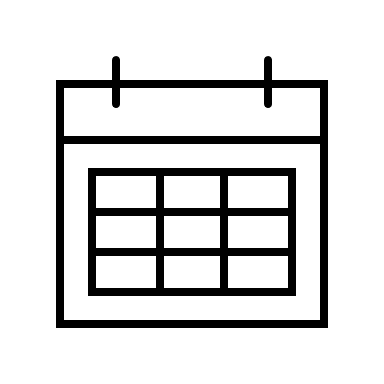 | Booked appointment: You have the option to schedule a meeting with a coach. |
| 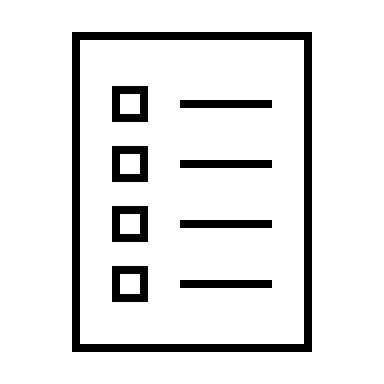 | Program: You can enroll in a structured program that begins with a digital kick-off meeting and includes ongoing coaching through digital messages. |

- What do you think could be the advantages and disadvantages of the different types of digital coaching features mentioned above?
- Is there anything we haven’t covered in today’s discussion about digital coaching that you would like to address or bring up?
